# T cells specific to multiple Bet v 1 peptides are highly cross-reactive toward the corresponding peptides from the homologous group of tree pollens

**DOI:** 10.3389/fimmu.2023.1291666

**Published:** 2023-11-22

**Authors:** Gitte Lund, Lars Harder Christensen, Jacob Ihlemann, Peter Sejer Andersen, Erik Wambre, Peter Adler Würtzen, Shashank Gupta

**Affiliations:** ^1^ Global Research Hoersholm, ALK, Hoersholm, Denmark; ^2^ Translational Immunology, Benaroya Research Institute at Virginia Mason, Seattle, WA, United States

**Keywords:** T-cells, cross-reactivity, birch homologous group, allergy immunotherapy, immunological mechanism, IgE

## Abstract

**Background:**

Allergens from Fagales trees frequently cause spring allergy in Europe, North America, and some parts of Asia. The definition of the birch homologous group, which includes birch (Bet v), oak (Que a), alder (Aln g), hazel (Cor a), hornbeam (Car b), beech (Fag s), and chestnut (Cas s), is based on high allergen sequence identity and extensive IgE cross-reactivity. Clinical effect was seen during the alder/hazel, birch, and oak pollen seasons after treatment with tree SLIT-tablets containing only birch allergen extract. Here, we characterize T-cell reactivity with respect to epitope specificities and cross-reactivity toward various Bet v 1 family members, (PR-10/group 1 major allergens). This cross-reactivity may be part of the immunological basis of clinical effect or cross-protection when exposed to birch homologous tree species.

**Method:**

T-cell lines were generated from 29 birch-allergic individuals through stimulation of peripheral blood mononuclear cells (PBMCs) with birch/Bet v or oak/Que a allergen extracts. T-cell responses to allergen extracts, purified group 1 allergens, and overlapping 20-mer peptides (Bet v 1, Aln g 1, Cor a 1, and Que a 1) were investigated by T-cell proliferation and cytokine production. Cross-reactivity was evaluated based on Pearson’s correlations of response strength and further investigated by flow cytometry using tetramer staining for homologous peptide pairs.

**Results:**

T-cell reactivity toward extracts and group 1 allergens from across the birch homologous group was observed for birch/Bet v as well as oak/Que a T-cell lines. T-cell lines responded to multiple Bet v 1 homologous peptides from Aln g 1 and Cor a 1 and a subset of Que a 1 peptides. Significant Pearson’s correlations between frequently recognized peptides derived from Bet v 1 and the corresponding peptides derived from alder, hazel, and oak strongly supported the T-cell cross-reactivity toward these allergens. Cross-reactivity between birch and birch homologous peptides was confirmed by pMHCII tetramer staining.

**Conclusion:**

T cells from birch tree pollen allergic individuals respond to multiple trees within the birch homologous group in accordance with the level of sequence homology between Bet v 1 family members, (PR-10 allergens) from these allergen sources, confirming the basis for clinical cross-protection.

## 1 Introduction

Allergic sensitization is characterized by the presence of allergen-specific IgE as well as Th2 cells activated by the same allergens that bind IgE. Cross-reactivity of IgE and T-cell receptors has immediate consequences for allergic symptoms and may also affect allergy immunotherapy (AIT) treatment (1, 2).

Exposure to pollen from birch and related trees (e.g., oak, hazel, and alder) is a common cause of allergic diseases such as rhinoconjunctivitis, and the prevalence of allergy to birch pollen was found to be as high as 18% in a worldwide study including 13 developed countries (3). Bet v 1 (PR-10) is the major allergen in birch (4, 5), and various birch-related trees contain PR-10-like molecules with high sequence identity to Bet v 1 (6–10) and very similar tertiary structures (11, 12). Moreover, several minor allergens have been characterized, which are also shared between some or all of these trees (13). IgE cross-reactivity and inhibition/depletion studies strongly suggest that the majority of the IgE binding components/epitopes are present in birch pollen extract and that the IgE is mainly directed toward group 1 major allergens (5, 14). The homology between the allergen sequences and their structure as well as the high level of IgE cross-reactivity demonstrated for the seven tree species (birch/Bet v, alder/Aln g, hornbeam/Car b, hazel/Cor a, oak/Que a, beech/Fag g/s, and chestnut/Cas s) is the reason for assigning them to the same homologous group (15, 16) termed the “birch homologous group” relevant for both diagnosis and AIT treatment (1, 2, 17).

Further knowledge about the immunological consequence of the allergen homology can be obtained from the reactivity of allergen-specific T cells and the cross-reactivity toward the PR10/group 1 allergen Bet v 1 and the homologous allergens in alder, hazel, hornbeam, and oak. This has also been investigated for T-cell lines and clones from individual birch pollen allergic patients (18–20). Investigations of cross-reactivity of Bet v 1-specific T-cell lines and clones toward alder, hazel, hornbeam, and oak (18, 19, 21) or of Cor a 1-specific T-cell lines (22) toward Bet v 1 all support that cross-reactivity toward these allergens also exists for T cells, but the number of individuals and allergens/epitopes investigated does not make it possible to conclude on the extent of this cross-reactivity.

The clinical consequence of the IgE and T-cell cross-reactivity is that most individuals who are allergic to birch pollen experience symptoms when exposed to pollen from the other members of the birch homologous group, which increases the burden of tree pollen allergy in terms of relevant seasons and regions (23).

The changes to the allergen-specific immune response during AIT with allergen extracts include induction of blocking non-IgE antibodies, as well as shifting the balance between Th1/Th2 and Treg cells (24). Thus, recognition of specific T-cell and B-cell epitopes appears to play a key role in mediating the clinical effects of the allergen extract used for treatment. Similarly, the treatment will affect allergens from other related species, recognized through cross-reactivity, as seen for the clinical cross-reactivity or cross-protection observed for grass SLIT-tablets (containing *Phleum pratense* extract) (25, 26) and ragweed SLIT-tablets (containing *Ambrosia artemisiifolia* extract) (20, 21), which are clinically effective in areas with seasonal exposure to multiple grass or ragweed species. This is fully supported by the IgE and T-cell cross-reactivity data for grass (25, 26) and ragweed (27, 28) as well as the cross-reactivity of treatment-induced IgG4 (25, 27). Clinical cross-protection has also been demonstrated for AIT with birch allergens extract (tree SLIT-tablets) in a chamber trial (1) and a field trial (2, 29).

In the current study, T-cell cross-reactivity is investigated in detail by evaluating the response toward individual T-cell epitopes in multiple allergic individuals (n = 32) and addressing the cross-reactivity in T-cell lines generated from most donors by stimulation with birch pollen extract or with oak pollen extract for comparison.

## 2 Methods

### 2.1 Design

A total of 32 individuals with IgE (ImmunoCAP) toward birch pollen allergen extract >0.7 kU/L were included in the study, and T-cell lines were established from peripheral blood mononuclear cells (PBMCs) through stimulation with birch/Bet v extract or oak/Que a extract. The specificity of the T-cell lines was mapped using allergen extracts, purified group 1 allergens, and 20-mer peptides spanning group 1 allergens from birch, alder, hazel, and oak. T-cell responses to hornbeam, beech, or chestnut were investigated to some extent and are included as **Supplementary Material** only. Tetramer analyses were performed to further characterize the extent of the T-cell cross-reactivity. IgE responses toward these seven allergen extracts were also determined, as shown in **Supplementary Table 1**. Informed consent was obtained from all subjects donating blood for the immunological tests. The collection of blood samples was approved either by the local ethics committees in Denmark (H-3-2014-129) or for subjects with HLA-DRB1*1501 haplotypes used for tetramer stain, recruited at the allergy clinic at Virginia Mason Medical Center (Seattle, WA) with approval from the Institutional Review Board of Benaroya Research Institute (IRB07109-605).

### 2.2 T-cell analyses

T cells from cryopreserved or freshly isolated PBMCs were expanded *in vitro* by culture with 5 µg/mL of either birch/Bet v or oak/Que a allergen extract as described previously (30), and specific responses were measured by thymidine incorporation on day 24 or later. In these assays, the T-cell lines were stimulated with individual peptides (2 µg/mL), purified allergens (2 µg/mL), allergen extracts (5–10 µg/mL), peptide pools (2 µg/mL), or controls (phytohaemagglutinin (PHA) or medium alone) in triplicates with T cells (3 × 10^4^/well) and also autologous PBMCs (2 × 10^4^/well, irradiated 3,000 rad) as antigen-presenting cells (APCs). Cells were cultured 3 days in a humidified atmosphere at 37°C and 5% CO_2_, followed by an 18-hour pulse with 0.5 µCi 3^H^-thymidine/well (PerkinElmer, Wellesley, MA, USA), and thymidine incorporation was determined by scintillation counting, counts per minute (CPM). The T-cell proliferation data are shown as relative magnitude for all statistically different responses with SI > 3 defined by CPM from the actual stimulated sample divided with CPM from the same cells with no stimulation (buffer only); all other values are shown as zero. The relative magnitude is calculated based on raw count from stimulation with the extract used for establishment and medium/baseline. The extract CPM subtracted baseline is set to 100, and the remaining baseline-corrected CPM are calculated relative to this. The raw count used is shown in **Supplementary Table 2**.

### 2.3 Cytokine measurements

Cell supernatants were harvested for cytokine measurement on day 2 post-stimulation, pooled from each triplicate stimulation, and measured as single measurements. The cytokines IFN-γ, IL-10, IL-13, IL-17A, and IL-5 were analyzed by Meso Scale assay using the U-plex kit U-plex (catalog no. Biomarker group 1: K15067L-2). The plate was read using Meso Scale Discovery (MSD). Cytokine concentrations were calculated in pg/mL as per the manufacturer’s protocol.

### 2.4 Allergen extracts and peptides

Experimental extracts of birch/Bet v, alder/Aln g, hazel/Cor a, hornbeam/Car b, oak/Que a, beech/Fag s, and chestnut/Cas s were made as follows: pollen from each species (10 g) was extracted in 100 mL of NH_4_HCO_3_ (0.125 mol/L, pH 8.3) at 5°C for 2 hours followed by dialysis (MW cutoff 3.5 kDa), filtration (0.2 mm), and freeze-drying. Endotoxin content of all extracts was below 20 EU/mg except for hazel/Cor a and chestnut/Cas s, which were below 100 EU/mg.

20-mer peptides (overlapping by 10 aa) covering amino acid sequences of Bet v 1, Aln g 1, Cor a 1, Que a 1, Car b 1, Fag s 1, and Cas s 1 were custom-made by GenScript (Piscataway, NJ, USA) with a purity of ≥95%. All cysteines were replaced with serine. The species of alder and beech differ between ImmunoCAP assays and T-cell experiments because Aln i and Fag g are the species available for ImmunoCAP, whereas group 1 major allergen sequences of Aln g and Fag s were reported in the literature and annotated in WHO/International Union of Immunological Societies (IUIS) allergen nomenclature databases.

The rationale for selecting Bet v 1.112 (or Bet v 1.2801), Aln g 1.0101, Cor a 1.0102, Car b 1.0109, Que a 1.0201, Fag s 1.0101, and Cas s 1.0101 was based on sequence availability, previous literature, and relative abundance (17, 31–33).

Group 1 allergens Bet v 1, Aln g 1, Cor a 1, and Que a 1 were purified from aqueous extract of pollen source materials. A series of size exclusion, ion exchange, and affinity chromatography were performed on the ÄKTA explorer 100 Air System to yield semi-pure preparations enriched with the relevant group 1 allergen. Enrichment of group 1 allergen in the individual preparations was confirmed by crossed immunoelectrophoresis and mass spectrometry. We did not make purified Cas s 1, Car b 1, and Fag s 1, and therefore, we used peptide pools of overlapping peptides covering the entire sequence for testing in T-cell assays.

IgE measurements were carried out using ImmunoCAP (Phadia 250, Thermo Fisher Scientific) as per the manufacturer’s instructions.

### 2.5 *Ex vivo* tetramer staining to determine the frequency of Bet v 1 and tree pollen homolog-specific CD4+ T cells

Bet v 1 and tree pollen homolog-specific CD4+ T cells (Cas a 1, Fag s 1, Que a 1, Aln g 1, and Cor a 1) were tracked as previously described in eight HLA-DRB1*1501-restricted birch pollen allergic individuals (34). Briefly, 20 million PBMCs in 200 μL T-cell culture medium were stained with 20 μg/mL fluorochrome-labeled tetramers (PE-labeled tetramers, PE-Cy7-labeled tetramers, and PE-CF594-labeled tetramers) for *ex vivo* combinatorial tetramer staining for 100 minutes, and empty tetramer staining was used as control (**Supplementary Figure S7C**). Cells were then washed and incubated with anti-PE magnetic beads (Miltenyi Biotec, Bergisch Gladbach, Germany) for 20 minutes at 4°C. Cells were next passed through a magnetic column (Miltenyi Biotec, Bergisch Gladbach, Germany). Bound PE-labeled cells were stained with a panel of antibodies of interest, including CD14 PerCP/Cy.5.5 (HCD14, BioLegend, San Diego, CA, USA), CD19 CD19 PerCP/Cy.5.5 (HIB19, BioLegend), CD45RA Alexa Fluor 700 (HI100, BD Biosciences, San Jose, CA, USA), and CD4 BUV737 (RPA-T4, BD Biosciences) for 20 minutes at room temperature. After staining, cells were stained with BD ViaProbes™ (BD Biosciences) for 10 minutes at 4°C before flow cytometry. Data were analyzed utilizing FlowJo (Tree Star, Ashland, OR, USA), gating on forward scatter/side scatter, and excluding CD14+, CD19+, and Viaprobe populations. The percentage of co-stained T-cell populations was determined relative to Bet v 1-specific T cells.

### 2.6 Statistical methodology

Responses to extracts and full-length allergens were compared: criteria for positive responses were set as previously described (30) on the basis of significant positive responses (Student’s t-test, p < 0.05) and stimulation index (SI) >3. All other measurements were set to zero. From here, the percentage of responding donors was calculated from each stimulus relative to the number of T-cell lines responding to the initial stimulus (birch or oak). The percentage of proliferative response relative to the initial stimulus (birch or oak) from each T-cell line was calculated and termed “relative magnitude” in order to normalize positive responses from T-cell lines. Differences in T-cell responses toward homolog extracts or allergens were evaluated by Friedman non-parametric ANOVA and Dunn’s multiple comparison rank sum test. T-cell cross-reactivity to individual peptide pairs was investigated by Pearson’s correlations for all lines with quantifiable response to Bet v 1 for birch lines (n = 29) and Que a 1 for oak lines (n = 22). GraphPad Prism 8 was used to make the statistical evaluation.

## 3 Results

### 3.1 IgE cross-reactivity

Serum samples collected from the individual birch-sensitized donors were analyzed for IgE specific to birch (Bet v) and birch homologous trees Aln I (alder), Cor a (hazel), Car b (hornbeam), Fag g (beech), Cas s (chestnut), and Que a (oak) by ImmunoCAP as presented in **Supplementary Table 1**. IgE sensitization and the correlation between IgE titers toward individual trees of the birch homologous group are shown in **Supplementary Figure S1**. These data confirm our previous findings from a larger cohort (17) and are described in **Supplementary Text 1.1**.

### 3.2 T-cell responses to extracts

**Figure 1:**
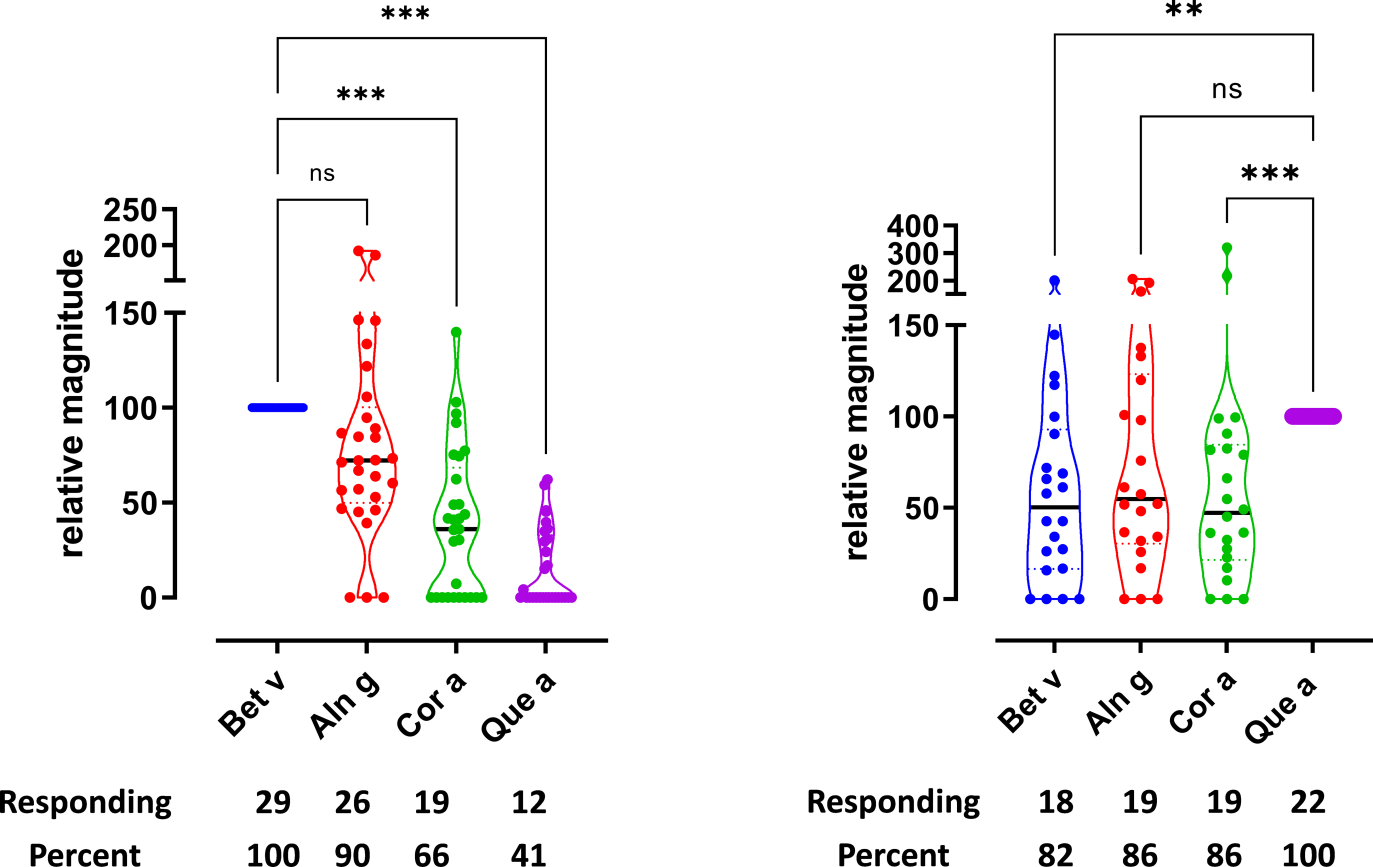
T-cell reactivity was observed toward allergen extracts from multiple trees. T-cell proliferation in response to allergen extracts. **(A)** Birch-specific T-cell lines, n = 29. **(B)** Oak-specific T-cell lines, n = 22. Relative magnitudes are indicated for all T-cell lines with SI > 3 or recoded to zero if SI < 3. Violin plots depict distribution of the data. Black lines show medians. Below are the number and percentage of positive responses with SI > 3. Differences evaluated by Friedman non-parametric ANOVA and Dunn’s multiple comparison rank sum test, p-value style NEJM, 0.12 (ns), 0.033 (*), 0.002 (**), <0.001 (***).

Simultaneous reactivity to different allergen extracts in a T-cell line generated by stimulation with a single allergen extract would suggest cross-reactivity. To address this, T-cell responses to various tree pollen extracts were investigated in T-cell lines generated from the PBMCs of each donor through stimulation with birch pollen extract (Bet v) or oak pollen extract (Que a). The proliferation data illustrate that birch T-cell lines as well as oak T-cell lines respond to allergen extracts from various tree species (**Figure 1**). The data are shown as relative magnitude, as described earlier in the Methods section. As reported previously (17), the majority of the birch T-cell lines respond to birch, alder, hazel, hornbeam, and beech (55%–100%), while chestnut and oak were recognized by 45% of the T-cell lines. In this study, we confirmed similar findings for birch, alder, hazel, and oak (**Figure 1A**). The strongest responses were observed for birch, alder, and hazel, whereas lower response strength was seen for oak. Hornbeam, chestnut, and beech were also evaluated for birch T-cell cross-reactivity, as shown in **Supplementary Figure S2A**. Birch T-cell lines responded to hornbeam, chestnut, and beech; however, the magnitude of response to chestnut and beech was lower compared to other birch homologs. The highest T-cell response was seen for alder and the lowest for chestnut (**Supplementary Figure S2A**). Supernatants from the T-cell lines stimulated with birch, alder, hazel, and oak were subjected to cytokine measurements. As shown in **Supplementary Figure S3**, the levels of cytokine IL-5, IL-13, and IFN-γ were found to follow the T-cell proliferation (**Figure 1A**) with significant differences seen primarily between birch and oak stimulations for the cytokines IL-5, IL-13, and IFN-γ, whereas IL-10 and IL-17a were not significantly different (**Supplementary Figure S3**).

For oak T-cell lines (**Figure 1B**), almost all lines responded to all extracts investigated (80%–100%), with oak inducing the strongest and very uniform responses. All other extracts, i.e., birch, alder, and hazel, induced lower responses on average and with a wider spread in the strength relative to the response to oak extract. Response toward hornbeam, chestnut, and beech was measured. The response to hornbeam and beech was found to be comparable; however, the response to chestnut was the highest (**Supplementary Figure S2B**). Supernatants from oak T-cell lines stimulated with birch, alder, hazel, and oak were further subjected to cytokine measurements. As shown in **Supplementary Figure S4**, the levels of cytokines were not found to be significantly different between tree extract stimulations and followed the T-cell proliferation data (**Figure 1B**). **Supplementary Figure S8** shows that the cross-reactivity is not observed in unrelated extracts for both birch T-cell lines and oak T-cell lines.

### 3.3 T-cell responses to purified group 1 allergens

**Figure 2:**
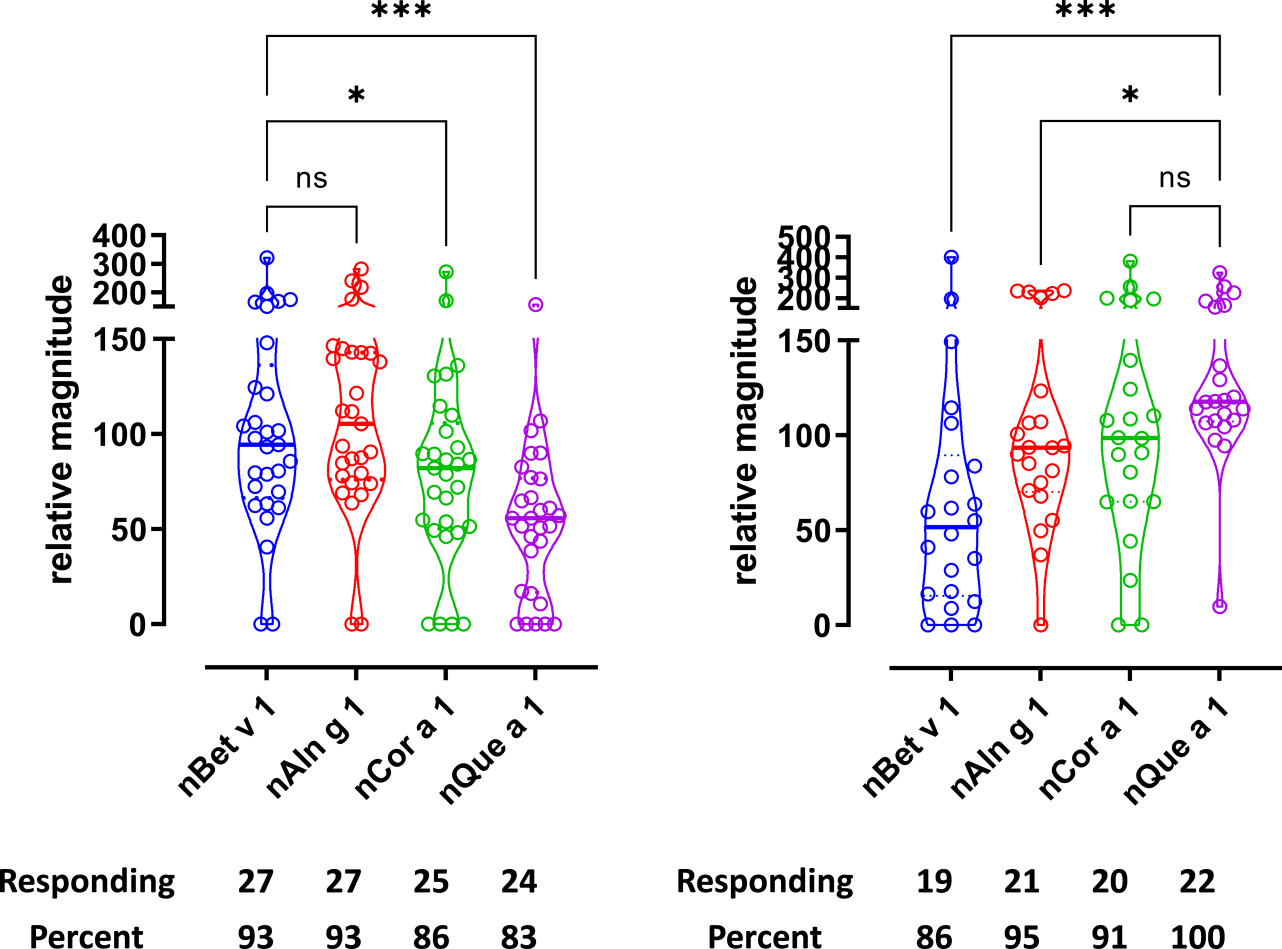
T-cell reactivity was observed toward PR-10/group 1 allergens from multiple birch homologous trees. T-cell proliferation in response to group 1 allergens. **(A)** Birch-specific T-cell lines, n = 29. **(B)** Oak-specific T-cell lines, n = 22. Relative magnitudes are indicated for all T-cell lines with SI > 3 or recoded to zero if SI < 3. Violin plots depict distribution of the data. Black lines show medians. Below are number and percentage of positive responses with SI > 3. Differences evaluated by Friedman non-parametric ANOVA and Dunn’s multiple comparison rank sum test, p-value style NEJM, 0.12 (ns), 0.033 (*), 0.002 (**), <0.001 (***).

Further support for T-cell cross-reactivity was obtained by investigating T-cell reactivity to homologous major allergens from individual tree species. Thus, T-cell responses to purified PR-10 like major allergens from birch, alder, hazel, and oak showed that nearly all birch T-cell lines (90%–100%) as well as all oak T-cell lines (85%–100%) responded (**Figure 2**). The strength of the responses in birch T-cell lines when stimulated with purified major allergens showed less variation among the donors than the responses to the extracts and ranked the major allergens Bet v 1=Aln g 1>Cor a 1>Que a 1 (**Figure 2A**). The strength of the response from oak T-cell lines when stimulated with purified major allergens showed more variation among donors with the following ranking: Que a 1 >Cor a 1>Aln g 1>Bet v 1 (**Figure 2B**). Car b 1, Cas s 1, and Fag s 1 were not available as purified allergens, and therefore, peptide pools of respective PR-10/group 1 allergens were tested for both birch and oak T-cell lines (**Supplementary Figure S5**). When tested with birch T-cell lines (**Supplementary Figure S5A**), Cas s 1 and Fag s 1 T-cell responses were comparable and were lower than Car b 1 response, whereas in oak T-cell lines (**Supplementary Figure S5B**), the responses to Car b 1, Cas s 1, and Fag s 1 were comparable. We did observe that the response to peptide pools was comparable to naturally purified group 1 allergens (nBet v 1 *vs.* Bet v 1 peptide pool and Que a 1 peptide pool *vs.* nQue a 1), suggesting that the majority of the T-cell epitopes have been covered in the peptide pool (**Supplementary Figure S5**), thereby generating comparable T-cell responses and validating the use of 20-mer peptide pools in absence of purified allergens.

### 3.4 Epitope mapping of group 1 allergens

**Figure 3:**
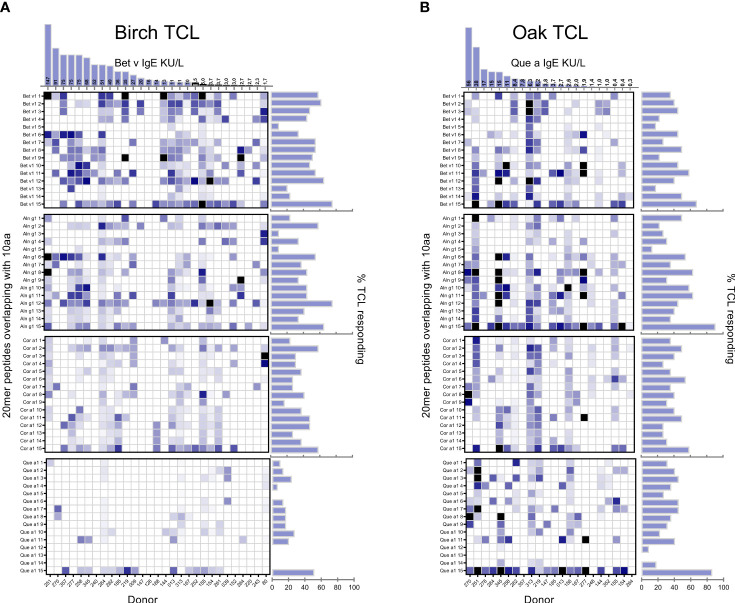
Peptide specificity of birch/Bet v **(A)** and oak/Que a **(B)** induced T-cell lines. % of responding T-cell lines is indicated for each peptide where peptides p01–15 are 20-mer peptides overlapping with 10 aa spanning the primary sequence of each of the group 1 allergens. Individual donor-derived T-cell lines are shown on the x-axis. The tree species are listed according to sequence homology to Bet v 1 with species listed toward the top showing higher levels of sequence homology. The color coding is based on relative magnitudes of T-cell proliferation with the response to the relevant allergen extract set to 100%. Right horizontal bars indicate percentage of TCL responding to indicated peptide with SI > 3 out of n = 29 birch and n = 22 oak T-cell lines. Columns at the top show serum IgE kU/L for birch/oak.

Next, we investigated the T-cell responses to individual peptides spanning the complete aa sequence of each of the four group 1 major allergens (Bet v 1, Aln g 1, Cor a 1, and Que a 1) to elucidate if the homologous peptides contain important T-cell epitopes relevant for cross-reactivity as indicated by the responses to allergen extracts and purified group 1 allergens. The amino acid sequence of the Bet v 1 homologs and the location of different peptides evaluated in the current study are illustrated in **Supplementary Figure S6**. The response frequency of the individual 20-mer peptides are aligned for birch/Bet v lines (**Figure 3A**) and for oak/Que a lines (**Figure 3B**) with the responses of individual donors and the strength of the responses depicted in the heat maps below each bar plot (**Figures 3A, B**). Responses to several homolog epitopes were observed from both birch and oak lines with respect to both frequency of response and magnitude, with the major difference in response to oak peptides from birch T-cell lines. Detailed comparison of epitopes is not the major scope of this paper but is discussed in **Supplementary Section 1.2**.

### 3.5 Cross-reactivity of T-cell responses—birch T-cell lines

**Figure 4:**
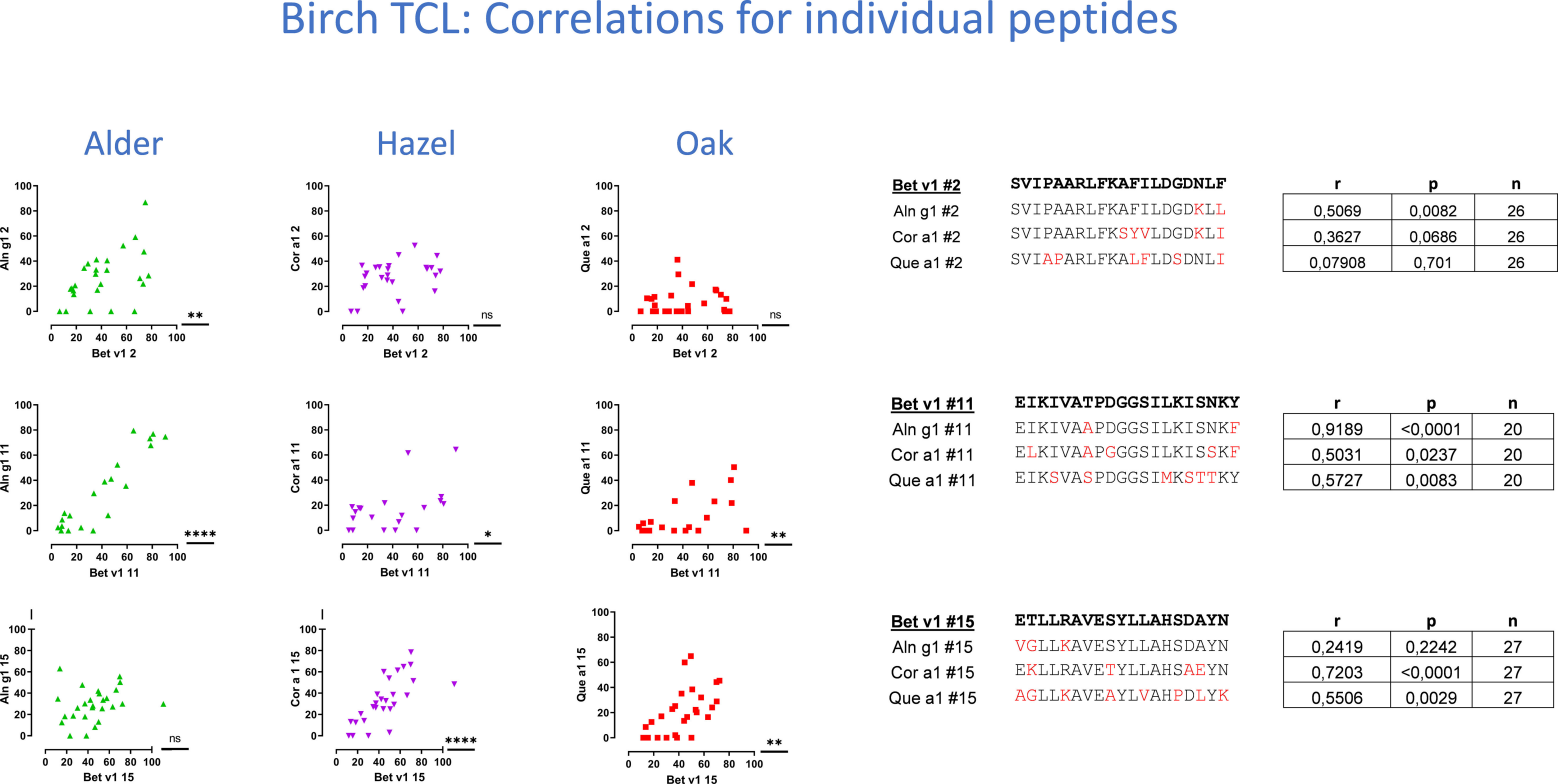
T-cell cross-reactivity illustrated by Pearson’s correlations plots for three example peptides of the 15 peptides investigated for each species. T-cell proliferation induced by Bet v 1 peptides is displayed on the x-axis, and proliferation induced by homologous peptides is displayed on the y-axis. Responses as shown by dots around the line of identity are suggested to be cross-reactive responses. T-cell lines with a positive response to any of the peptides were included in the determinations of Pearson’s correlation. Pearson’s r and p-values and n are displayed in tables to the right, next to amino acid sequences ordered according to the level of sequence homology between Bet v 1 and each of the group 1 allergens.

The level of cross-reactivity of Bet v 1 epitopes relative to the homologous epitopes of the other group 1 allergens was quantified using Pearson’s correlation r-values as a measure of cross-reactivity (examples in **Figure 4**, summarized in **Table 1A**). This revealed a significant correlation of T-cell responses to peptides #7_(61-80)_ and #11_(101-120)_ of Bet v 1 and all three homologous group 1 allergens. For most peptides, T-cell reactivity was quite heterogeneous among the homologous species, suggesting that particular amino acid substitutions affected T-cell activation negatively. Other epitopes showed T-cell cross-reactivity restricted to only one or two species, which included several Aln g 1 peptides alone or in combination with either the homologous Cor a 1 or Que a 1 peptides. This leads to an overall ranking of cross-reactivity: Aln g 1>Cor a 1>Que a 1 in line with the T-cell responses to full-length group 1 allergens and allergen extracts. Cross-reactivity restricted to only Bet v 1, Cor a 1, and Que a 1 was seen for peptide #15_(141-159)_. However, many T-cell lines did respond to peptide #15_(141-159)_ from Aln g 1 as well, but a significant correlation was not observed (**Figure 4**, lower right corner).

**Table 1:** Summary of cross-reactivity evaluated by Pearson’s correlations.

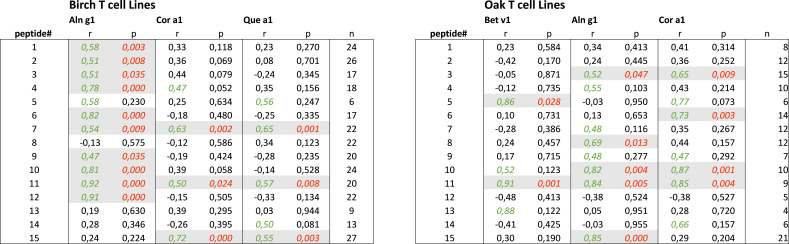	

Left, Table 1A, shows the reactivity of Aln g 1, Cor a 1, and Que a 1 peptides in the birch T-cell lines. Right, Table 1B, shows the peptide responses in oak T-cell lines. r, p, and n representing T-cell lines correspond to the peptide shown. Significant p-values <0.05 are shown in red italics and r > 0.45 in c significant correlations with p < 0.05 and r > 0.45.

### 3.6 Cross-reactivity of T-cell responses—oak T-cell lines

**Figure 5:**
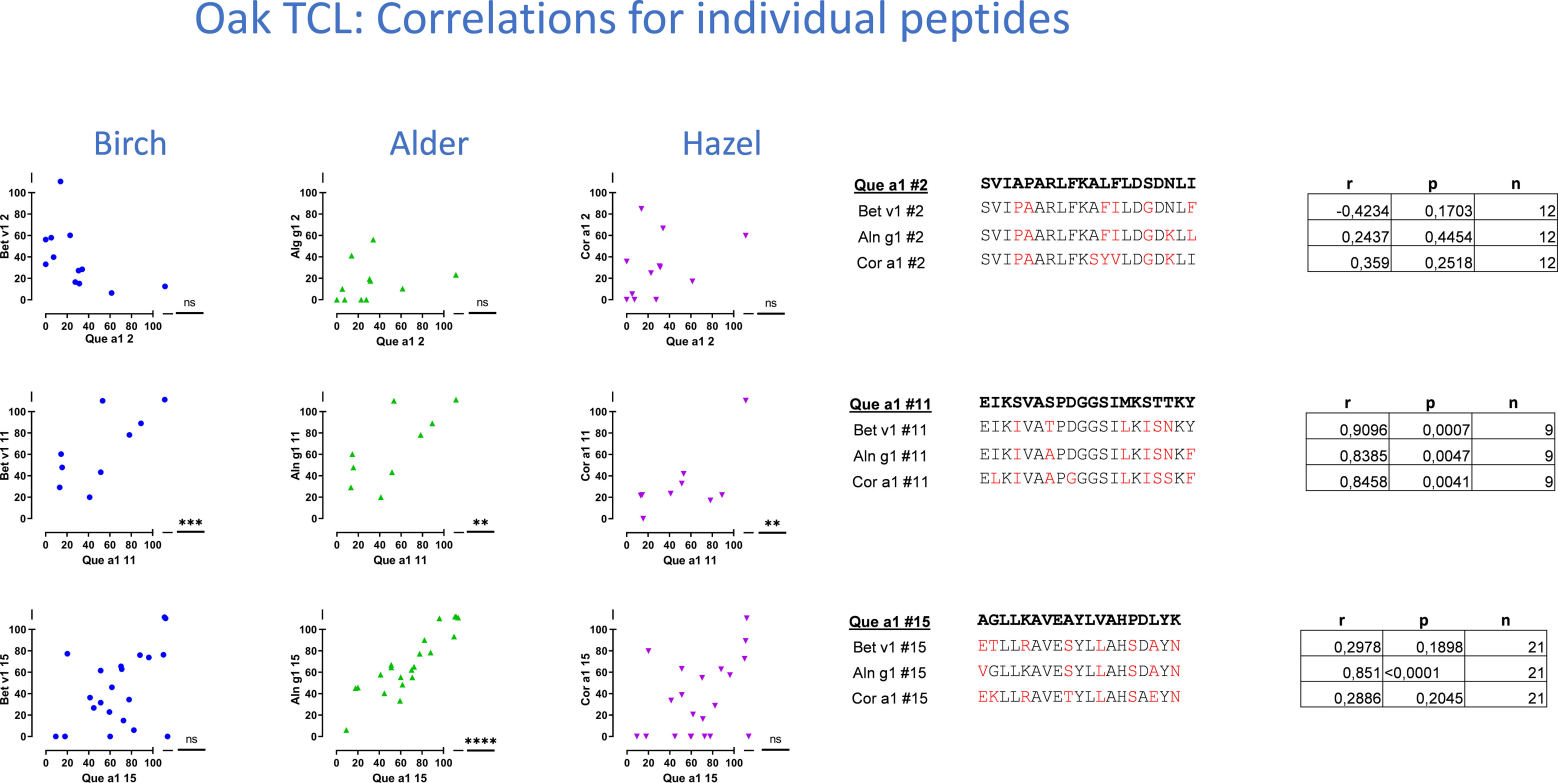
T-cell cross-reactivity illustrated by Pearson’s correlations plots for three example peptides of the 15 peptides investigated for each species. T-cell proliferation induced by Que a 1 peptides is displayed on the x-axis, and proliferation induced by homologous peptides is displayed on the y-axis. Lines with a positive response to any of the peptides were included in the determinations of Pearson’s correlation. Pearson’s r p and n are displayed in table to the right, next to amino acid sequences ordered according to the level of sequence homology between Que a 1 and each of the group 1 allergens.

For oak T-cell lines, cross-reactivity between Que a 1 epitopes and homologous peptides was most frequently observed for Aln g 1 peptides (5/15), followed by Cor a 1 peptides (4/15) and Bet v 1 peptides (2/15) (**Table 1**). For peptides #3_(21-40)_ and #10_(91-110)_, a significant correlation of T-cell responses was seen for Que a 1 and Aln g 1 as well as Que a 1 and Cor a 1. In a single case, a significant correlation of T-cell responses to a Que a 1 peptide (peptide #11_(101-120)_) and the corresponding peptides from all 3 homologous group 1 allergens was observed. For peptide #7, a significant correlation was seen for birch T-cell lines, but not for oak T-cell lines. This clear difference in the level of cross-reactivity between Bet v 1 peptides and peptides from Cor a 1 or Aln g 1 was not seen for the responses to the full-length allergens or the allergen extracts. However, it was in line with the evaluation of peptide cross-reactivity based on the birch T-cell lines, also suggesting a lower level of cross-reactivity for Bet v 1 and Que a 1 peptides. Cross-reactivity restricted to only Que a 1 and Aln g 1 was seen for peptide #15_(141-159)_. However, in this case, many oak T-cell lines did respond to peptide #15_(141-159)_ from Cor a 1 and Bet v 1 as well, but significant correlations were not observed (**Figure 5**, lower right table, and **Table 1B**).

Overall, quantification of T-cell cross-reactivity in birch and oak T-cell lines by Pearson’s correlation supported that significant cross-reactivity to homologous peptides from Bet v 1, Aln g 1, Cor a 1, or Que a 1 was observed for multiple peptide pairs, albeit with less cross-reactivity seen for Bet v 1/Que a 1 peptide pairs.

### 3.7 Evaluation of T-cell cross-reactivity by pMHCII tetramer

**Figure 6:**
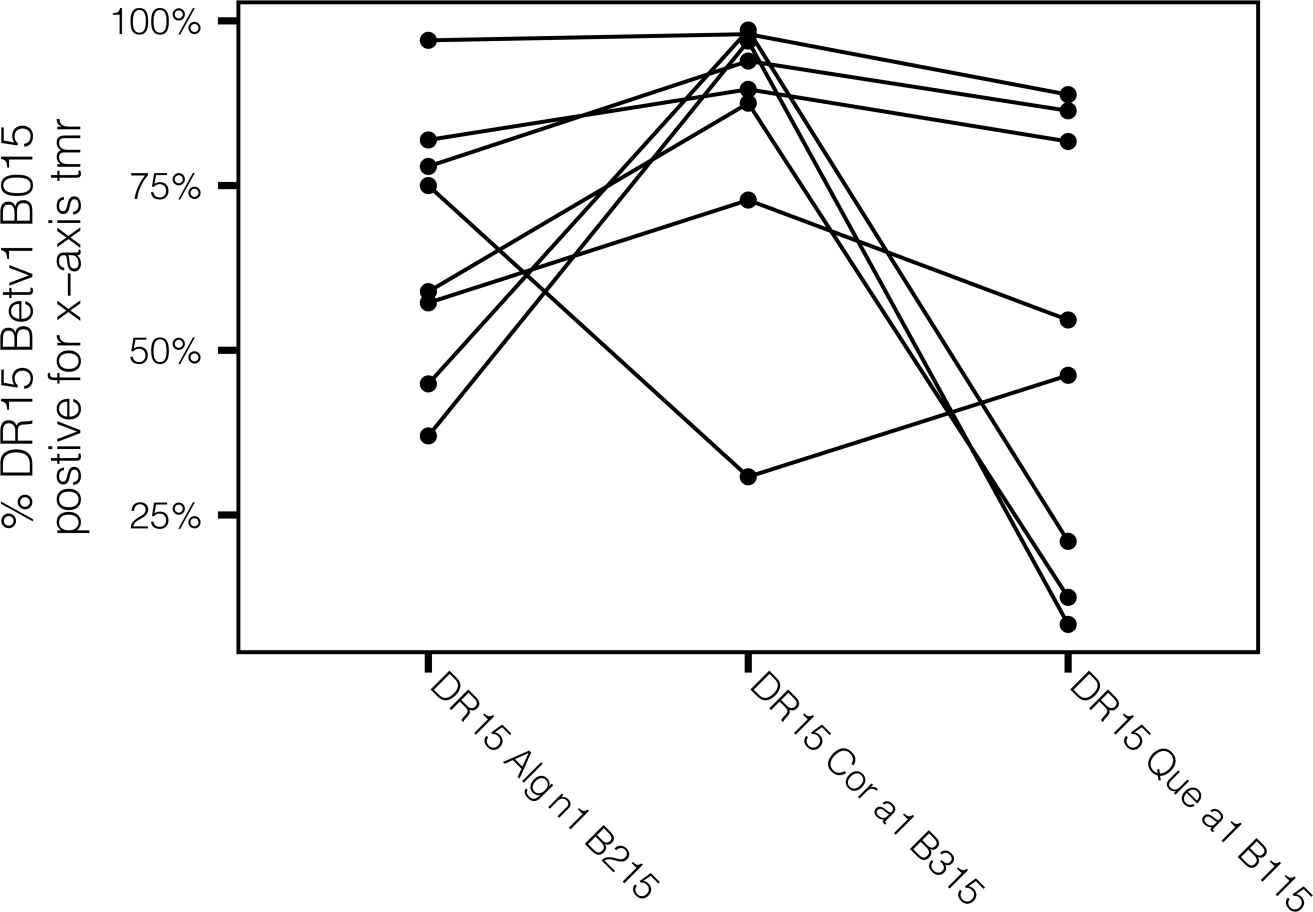
Group 1 tree pollen-specific CD4+ T cells exhibit varying degrees of cross-reactivity. Comparison of percentages of HLA-DRB1*1501-restricted cross-reactive T cells for Group 1 tree pollen homologs from birch-allergic individuals (n = 8). Each dot depicts percentage of co-stained T cells relative to HLA-DRB1*1501-restricted Bet v 1 peptide #15_(141-159)_-specific CD4+ T cells.

T-cell cross-reactivity was evaluated by co-staining assays utilizing pMHCII tetramers loaded with Bet v 1-derived peptide #15 and tetramers with homologous tree pollen epitopes. These experiments focus on HLA-DRB1*1501 because this allele was prevalent in our cohort of subjects with birch pollen allergy. The cross-reactive patterns for the epitopes are summarized in **Figure 6** and **Supplementary Figure S7** and listed in **Supplementary Table 3**. Three different cross-staining patterns were identified based on the proportion of cross-reactivity among Bet v 1-specific T cells. The majority of Bet v 1 peptide #15_(141-159)_-specific T cells tracked by HLA-DRB1*1501 tetramer cross recognize the Aln g 1 peptide #15_(141-159)_ and Cor a 1 peptide #15_(141-159)_ epitopes in the corresponding region and were identified as full-scale cross-reactive epitopes (**Figure 6**). However, HLA-DRB1*1501-restricted Bet v 1 homologous antigenic-epitopes Que a 1 peptide #15_(141-159)_ (**Figure 6**) as well as Cas a 1 peptide #15_(141-159)_ and Fag s 1 peptide #15_(141-159)_ (**Supplementary Figure S7**) were identified as partial cross-reactive T-cell epitopes. Indeed, these epitopes showed high cross-reactivity (co-stained higher than 25%) in some but not all tested allergic individuals. Alder 6/8, hazel 7/8, and oak 4/8 showed cross-reactivity >50%. These data suggest that the patterns of cross-reactivity among tree pollen allergen homolog epitopes are diversified and follow the sequence homology, which is in line with the cross-reactivity data obtained from T-cell line stimulations as shown in **Figure 4**.

## 4 Discussion

The current investigation aimed to determine the involvement of individual T-cell epitopes in the cross-reactivity to group 1 major allergens from the birch homologous group members birch, alder, hazel, and oak. The homologous groups for allergy diagnosis and AIT have been established on the basis of sequence and structural homology for the major allergens as well as IgE cross-reactivity toward these allergens (15). For some homologous groups, T-cell responses have been addressed as well, and the data demonstrate that T-cell cross-reactivity is involved in the similar immune responses observed for the homologous allergens in grass (20, 25, 35) and ragweed (28, 36).

For the trees belonging to the birch homologous group, the importance of PR-10/group 1 major allergens for T-cell recognition was indicated in the present investigation by the finding that T-cell responses to Bet v 1 were comparable or even higher in strength than responses to the birch pollen extract. Similar data were obtained for the alder, hazel, and oak extracts and corresponding group 1 allergens, pointing toward the importance of the Bet v 1 homologs for T-cell activation by each of the tree pollens investigated.

Multiple T-cell epitopes in Bet v 1 have been identified (18, 19, 37, 38), and early work indicated that the diversity of the response would make it difficult to design peptide AIT for birch allergy (19). However, the center (Bet v 1_77-92_) and the C-terminal part (Bet v 1_142-156_) of the molecule have been suggested to contain dominant epitopes (19, 39), with the latter being identified with high frequency in almost all studies conducted to date. The current data confirm these findings and suggest that additional epitopes are important for the T-cell response to Bet v 1 because most peptides were recognized by approximately half of the birch T-cell lines investigated.

The use of T-cell clones in the early studies [reviewed by van Neerven (37)] made it clear that T-cell cross-reactivity led to the parallel recognition of PR-10/group 1 allergens from birch, alder, and hazel. Many amino acid substitutions between species or between individual isoforms did not affect T-cell activations, but some differences completely abrogated T-cell activation. However, the extent of the cross-reactivity differed between studies when investigating one to two clones from each individual in small groups of patients (19, 20) or multiple clones from two to three patients (18, 19). The current data are derived from 51 T-cell lines from more than 20 donors generated by stimulation with either birch or oak extracts. They demonstrate that the T-cell cross-reactivity is a general phenomenon involving multiple epitopes in most tree pollen-allergic individuals and that the initial stimulation in culture has only little influence on the extent of the cross-reactivity observed. In general, multiple peptides were involved in the T-cell activation. Across the four homologous group 1 allergens investigated, the proportion of peptides recognized by the birch T-cell lines and the strength of the responses were in accordance with the level of sequence homology (Bet v 1>Aln g 1>Cor a 1>Que a 1). In addition, when investigating the cross-reactivity further using Pearson’s correlation coefficients between the strength of T-cell responses for each peptide pair to quantify the level of cross-reactivity as described previously for ragweed allergens (28), the number of cross-reactive peptides also reflected the sequence homology of the group 1 allergens.

The dependence on sequence homology was not so obvious for the oak T-cell lines, responding similarly to Bet v 1/Aln g 1/Cor a 1. T-cell responses to Aln g 1, Cor a 1, and Bet v 1 resulted in significant Pearson’s correlations for five, four, and two peptide pairs, respectively, although the lower number of oak T-cell lines investigated may compromise the quantification approach. While the PR-10/group 1 allergen with the least sequence identity (Bet v 1) was identified as the least cross-reactive, these findings also show that oak T-cell responses to the three homologous allergens investigated were quite similar. This high level of similarity in responses to Bet v 1/Aln g 1/Cor a 1 could suggest that oak-specific T cells in Danish donors represent a subset of the T-cell population that recognizes group 1 major allergens from all of these tree pollens with similar frequencies. The degree of cross-reactivity was confirmed when investigating cytokine production from the T-cell lines with similar patterns for Th1 and Th2 cytokines.

A very recent paper by Polak et al. (40) investigated T-cell responses from eight Aln g allergic individuals through the generation of T-cell lines by stimulation with various recombinant Bet v 1 homologous allergens and described their reactivity to Bet v 1 peptides. The data seem to support that T-cell responses are less cross-reactive than might have been expected. However, the T-cell lines generated by rAln g 1 that were characterized in detail recognized a limited number of important epitopes with only two regions considered dominant (more than 50% of patients responding). This is very much in contrast to the T-cell recognition of multiple epitope-containing peptides from most group 1 allergens observed in the current report. Various differences in methodology between the two studies, recombinant allergens *vs.* allergen extracts, 20-mer *vs.* 12-mer peptides, geographical origin of the donors, and T-cell culture procedures make it difficult to compare the results directly. Examples of T-cell lines with little cross-reactivity were also found when investigating T-cell responses to Cor a 1.04, which is predominant in hazelnut (22), and such lines were also seen in a minor subset of donors in the current study. An additional observation by Polak et al. was that the dominant Bet v 1 T-cell epitope (Bet v 1_141-155_) was the main reason for cross-reactivity to Bet v 1 but that this epitope was not leading to cross-reactivity of the rAln g 1 T-cell lines toward Bet v 1. In the present data set, a significant correlation was not observed for Bet v/Aln g responses to the C-terminal peptide even though several individual T-cell lines did show very similar levels of response.

Peptide-MHCII tetramer analyses of Bet v 1 (41) and Aln g 1-specific T-cell responses (42) have identified the homologous C-terminal peptide (Bet v 1_141-155_/Aln g 1_142-154_) as important T-cell epitopes in both species, further supporting the cross-reactivity as such. In-depth characterization of the Aln g 1-specific T cells specific for the C-terminal epitope led to the initial identification in humans of terminally differentiated inflammatory Th2 cells or Th2A cells, suggesting that this T-cell phenotype exclusively found in allergic individuals and shown to be a target of AIT (43) is part of a cross-reactive immune response toward Bet v 1 homologous allergens. In the current study, T cells with the same peptide specificities as these Aln g 1-specific Th2A cells were also found, and especially, the (C-terminal) peptide #15_(141-159)_ was found to be frequently recognized in all four allergens investigated, regardless of whether the T-cell lines were generated by stimulation with birch or oak allergen extract. The tetramer staining data illustrate the T-cell cross-reactivity and further confirm the cross-reactivity within the birch homologous group at the epitope level. The majority of Bet v 1 peptide #15_(141-159)_-specific T cells were highly cross-reactive toward peptide #15_(141-159)_ from hazel and alder, whereas partial cross-reactivity was observed with peptide #15_(141-159)_ from sweet chestnut, beech, and oak. The tetramer data reassuringly reflect the level of cross-reactivity that was observed in the T-cell proliferation assay, further validating this approach. Some donors may be sensitive to minor differences in the peptide sequences, so the cross-reactivity cannot be taken for granted. Overall, the degree of cross-reactivity relates to the level of sequence homology between PR-10/group 1 allergens from these allergen sources.

In our previous study, we investigated the immune modulating effect of SQ tree SLIT-tablet by addressing changes in a) serum levels of cross-reactive IgE and IgG4, b) the functionality of these antibodies, and c) link to the clinical effect demonstrated in EEC sessions and during the alder/hazel, birch, and oak pollen seasons (1, 17, 29). The current data support the concept of the birch homologous groups and further expand on the T-cell cross-reactivity and its possible involvement in AIT. Several 20-mer peptides from the group 1 allergens of alder, hazel, and oak tree species investigated in detail harbored epitopes that met the criteria for cross-reactivity set up by Pearson’s correlation. Some donors did show species selectivity for some epitopes, and the strength of the responses may vary. However, the finding that stimulation of birch and oak T-cell lines with extracts as well as purified allergens activated the vast majority of the lines investigated strongly indicates that the existing T-cell responses toward homologous species will be activated and thereby modulated by AIT based on an individual tree pollen extract. Further studies in AIT-treated individuals are needed to pinpoint the importance of individual epitopes and establish the threshold for the strength/coverage needed to adequately modulate T-cell responses to homologous species and the possible induction of Th1/Treg responses by epitope spreading (44). The current data may also support that inflammatory Th2A cells are involved in the cross-reactive T-cell response. The #15_(141-159)_ epitope for alder, which was a part of the original investigations addressing Th2A cells (42), did not meet the criteria for being a significantly cross-reactive epitope, but the Th2A phenotype was also found in other allergies and are not believed to be restricted to individual epitopes, so cross-reactivity related to other epitopes may contribute to the modulation of this T-cell phenotype during AIT.

## Data availability statement

The original contributions presented in the study are included in the article/**Supplementary Material**. Further inquiries can be directed to the corresponding author.

## Ethics statement

The studies involving humans were approved by Local ethics committees Denmark (H-3-2014-129) and approval from Institutional Review Board of Benaroya Research Institute (IRB07109-605). The studies were conducted in accordance with the local legislation and institutional requirements. The participants provided their written informed consent to participate in this study.

## Author contributions

GL: Data curation, Formal Analysis, Investigation, Methodology, Validation, Writing – original draft, Writing – review & editing. LC: Formal Analysis, Methodology, Supervision, Writing – review & editing. JI: Methodology, Writing – review & editing. PA: Investigation, Methodology, Supervision, Writing – review & editing. EW: Formal Analysis, Investigation, Methodology, Writing – review & editing. PW: Conceptualization, Data curation, Investigation, Methodology, Writing – original draft, Writing – review & editing. SG: Conceptualization, Data curation, Formal Analysis, Investigation, Methodology, Project administration, Resources, Supervision, Visualization, Writing – original draft, Writing – review & editing.
