# Supplementary figures and images for: T cells specific to multiple Bet v 1 peptides are highly cross-reactive toward the corresponding peptides from the homologous group of tree pollens
Source: Front Immunol. 2023 Nov 22;14:1291666. doi: 10.3389/fimmu.2023.1291666 (PMC10702988; doi:10.3389/fimmu.2023.1291666)

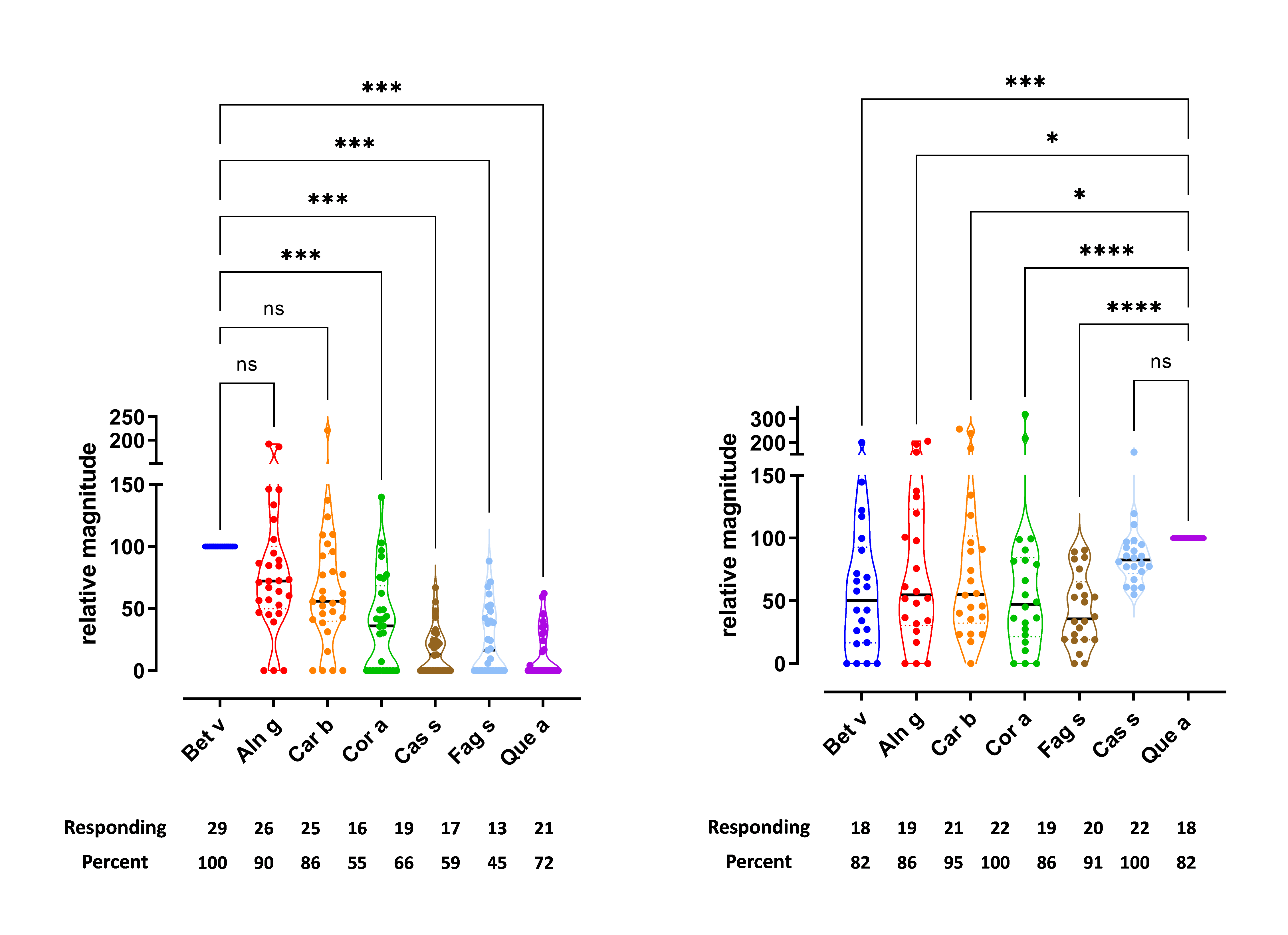

Supplement: Supplementary file 2 [file Image_2.tif]

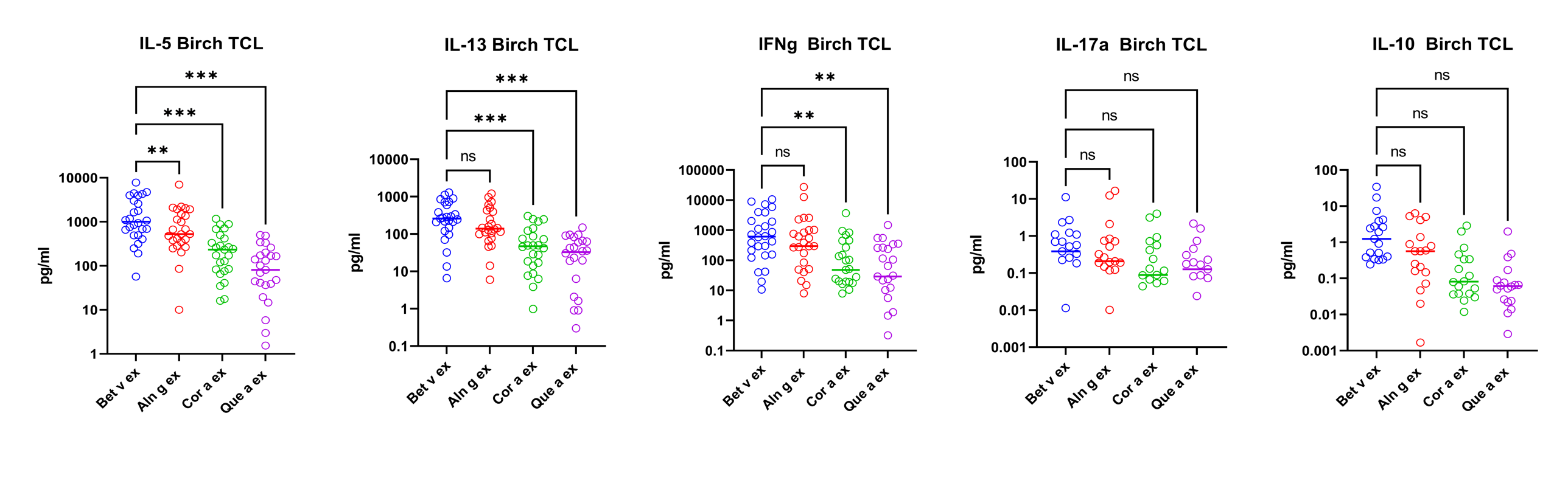

Supplement: Supplementary file 3 [file Image_3.tif]

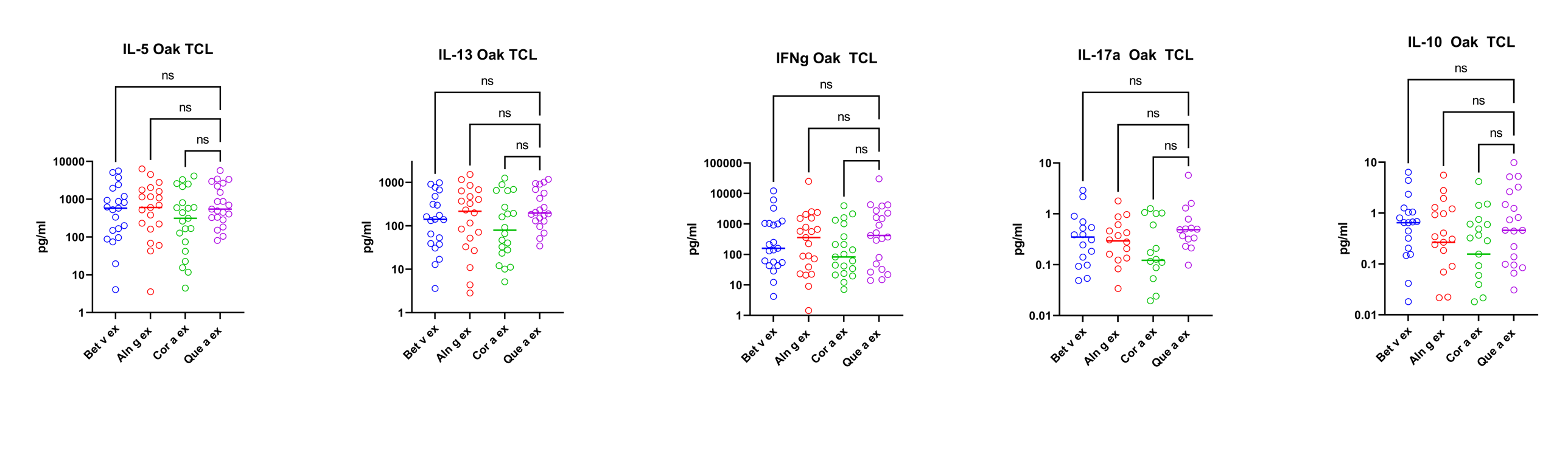

Supplement: Supplementary file 4 [file Image_4.tif]

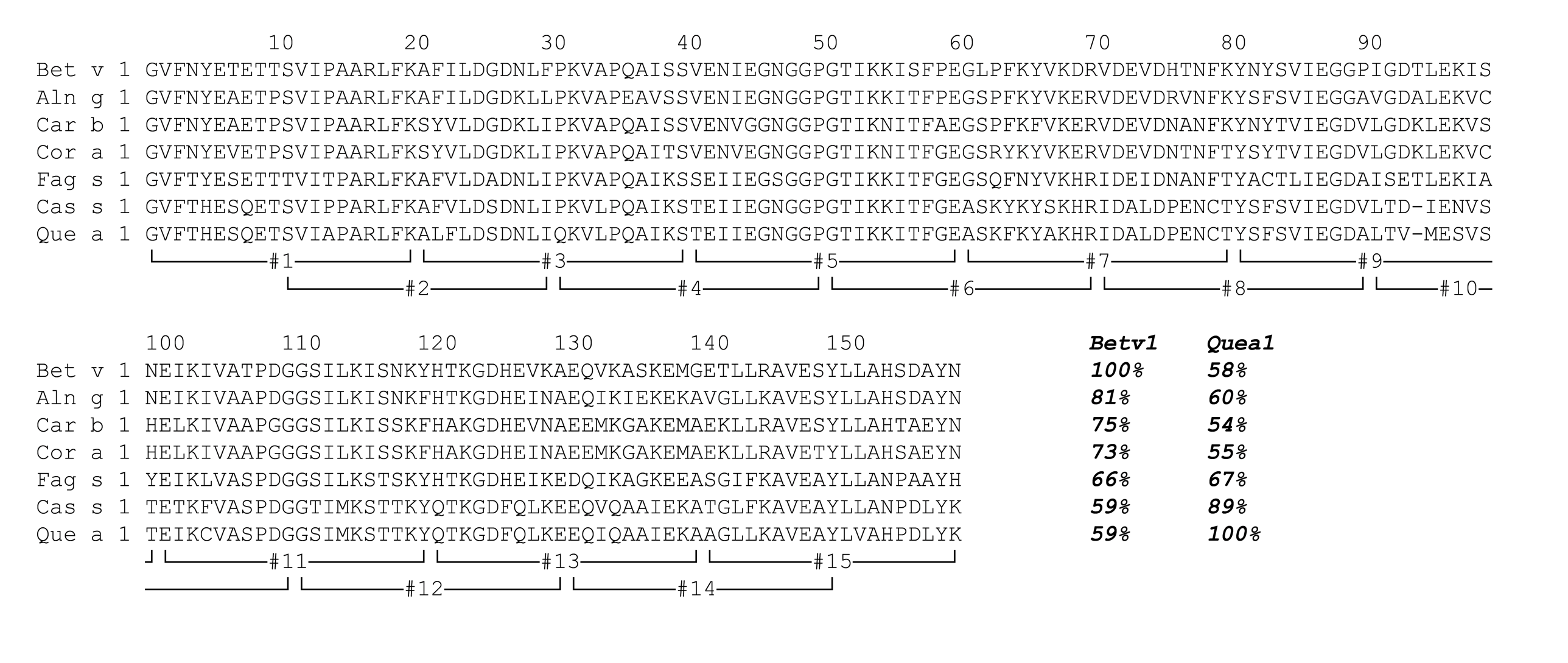

Supplement: Supplementary file 6 [file Image_6.tif]

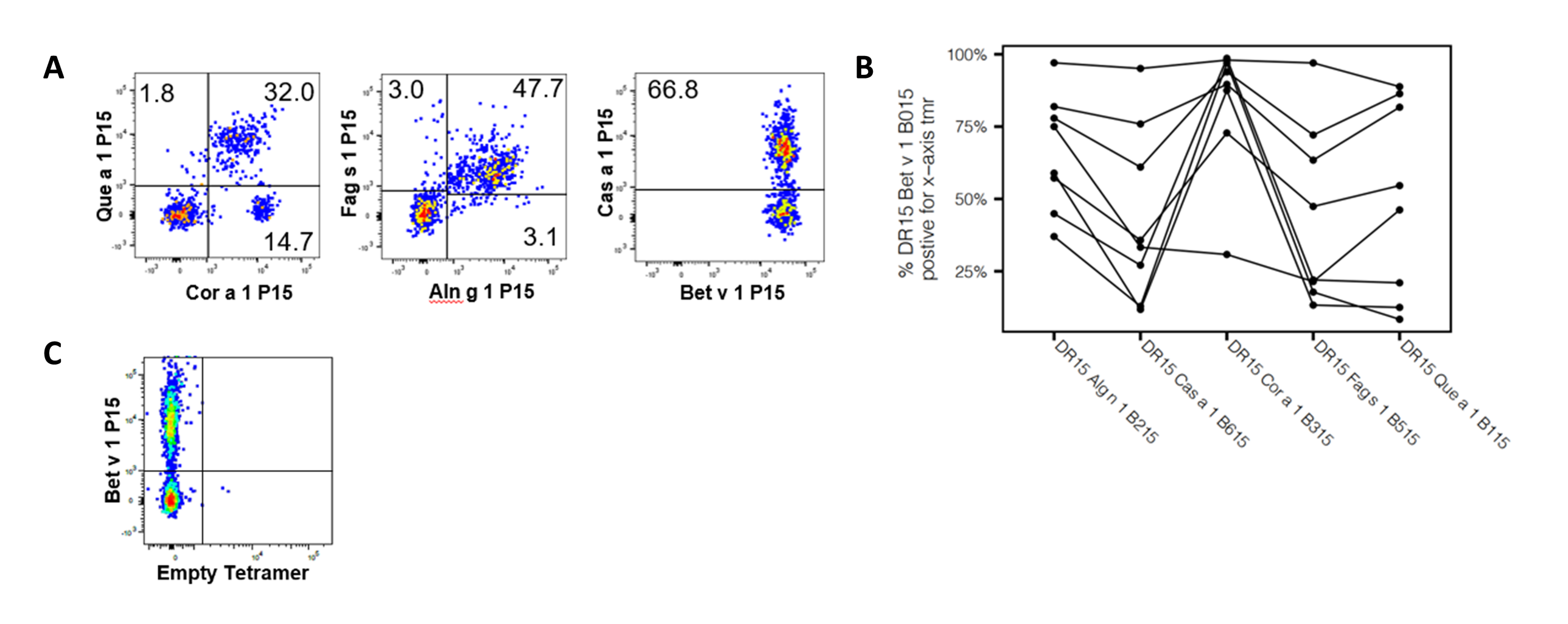

Supplement: Supplementary file 7 [file Image_7.tif]
