# Supplementary material for: T cells specific to multiple Bet v 1 peptides are highly cross-reactive toward the corresponding peptides from the homologous group of tree pollens
Source: Front Immunol. 2023 Nov 22;14:1291666. doi: 10.3389/fimmu.2023.1291666 (PMC10702988; doi:10.3389/fimmu.2023.1291666)
Supplement: Supplementary file 9 [file Table_1.pdf]

Donors	Bet v	Aln i	Cor a	Car b	Fag g	Cas a	Que a
A	27,01	16,22	7,81	8,50	8,89	0,35	4,04
B	11,42	12,72	5,70	6,64	3,77	0,35	2,67
C	3,05	2,06	1,65	1,73	1,44	0,35	0,35
D	0,35	0,35	0,35	0,35	0,35	0,35	0,35
E	16,02	12,69	5,63	6,15	4,73	0,35	2,82
F	12,81	10,01	6,07	7,01	7,49	0,35	1,05
G	19,99	13,11	4,61	3,52	8,66	0,35	3,79
H	3,01	2,19	2,10	1,68	1,90	0,35	0,78
I	3,69	3,39	1,47	1,55	0,90	0,35	0,35
J	5,08	2,23	0,91	0,99	1,59	0,35	0,35
K	14,45	10,69	10,92	11,28	11,85	0,35	2,60
L	10,44	6,28	2,75	2,07	5,21	0,35	2,02
M	35,54	20,65	9,11	9,83	13,18	0,35	3,70
N	13,85	6,40	3,42	3,09	2,94	0,35	1,36
O	2,66	2,52	1,75	0,57	2,18	0,35	0,85
P	51,72	30,15	23,23	16,33	20,51	0,98	16,34
Q	2,31	1,50	1,04	1,15	0,86	0,35	0,35
R	2,59	2,20	1,73	1,70	1,32	0,35	1,41
S	147,24	143,04	91,65	91,82	36,90	0,35	37,83
T	74,61	28,43	6,78	18,46	4,38	0,35	10,93
U	3,65	2,76	1,86	1,96	1,22	0,35	0,91
V	2,70	1,80	0,79	0,38	0,90	0,35	0,35
W	90,81	105,16	64,21	55,30	76,64	25,51	55,96
X	74,87	43,96	13,04	12,45	9,31	0,35	1,91
Y	67,05	37,48	26,32	34,76	21,24	0,35	17,12
Z	48,76	27,71	20,92	18,35	21,54	0,35	8,42
AA	51,28	35,65	23,67	23,35	23,63	0,35	14,84
AB	68,23	47,27	27,78	32,58	31,10	0,35	14,69
AC	1,69	1,36	1,40	0,98	1,31	0,35	1,24
AD	8,54	4,40	1,36	3,82	2,18	0,35	1,01
AE	35,15	22,51	17,55	20,37	14,64	0,35	6,17
AF	75,09	54,68	43,31	32,34	29,90	0,35	7,84
AG	10,61	8,65	5,24	4,48	9,67	1,85	6,26

Supplementary Table 1: *The IgE-sensitization of all donors towards birch/Bet v, alder/Aln i, hazel/Cor a, hormbeam/Car b, beech/Fag g, chestnut/Cas a and oak/Que a was investigated by immunoCAP and the values are shown as kU/L. Lower level of quantification (LLQ) is 0.7 kUA/L (dotted lines) and all values below this value were recoded to 0.35 kUA/L*
