# Supplementary material for: T cells specific to multiple Bet v 1 peptides are highly cross-reactive toward the corresponding peptides from the homologous group of tree pollens
Source: Front Immunol. 2023 Nov 22;14:1291666. doi: 10.3389/fimmu.2023.1291666 (PMC10702988; doi:10.3389/fimmu.2023.1291666)
Supplement: Supplementary file 10 [file Table_2.pdf]

Average Raw values T-cell lines, CPM								
Birch T-cell lines					Oak T-cell lines			
Donor	Birch ex	Medium	SI		Donor	Oak ex	Medium	SI
A	26402	3851	7					
B	68215	3442	20		B	66133	10013	7
C	33165	5867	6					
E	68382	11167	6					
F	75301	9381	8		F	55380	2112	26
G	69399	12902	5		G	91861	18597	5
H	72511	10698	7					
I	37753	826	46		I	18777	2357	8
J	32785	1171	28		J	62274	6864	9
					K	26742	1406	19
L	51098	1524	34		L	65923	5118	13
M	63748	3950	16		M	61923	5907	10
N	104384	18287	6					
P	36923	4940	7					
Q	37290	6778	6					
					R	53415	4442	12
S	18300	1655	11		S	16015	1314	12
T	74510	3243	23		T	72518	4047	18
U	87635	5665	15					
V	21586	2592	8		V	5884	218	27
W	28751	2646	11		W	20114	787	26
X	38904	3032	13		X	20949	2637	8
					Y	79907	12677	6
Z	37182	1299	29		Z	40030	1563	26
AA	35068	597	59		AA	49266	5582	9
AB	46597	1946	24		AB	8248	220	38
AC	18462	407	45					
AD	27648	4848	6		AD	21895	1332	16
					AE	31665	1454	22
AF	51575	11479	4		AF	57034	14222	4
AG	57249	959	60		AG	33014	670	49
