# Supplementary material for: T cells specific to multiple Bet v 1 peptides are highly cross-reactive toward the corresponding peptides from the homologous group of tree pollens
Source: Front Immunol. 2023 Nov 22;14:1291666. doi: 10.3389/fimmu.2023.1291666 (PMC10702988; doi:10.3389/fimmu.2023.1291666)
Supplement: Supplementary file 11 [file Table_3.pdf]

Donor	HLA	Tetramer	Percentage pos
AA	DR15	DR15 Casa1 B615	27.1
AA	DR15	DR15 Fags1 B515	22
AA	DR15	DR15 Cora1 B315	98.6
AA	DR15	DR15 Algn1 B215	44.9
AA	DR15	DR15 Carb1 B415	99.1
AA	DR15	DR15 Quea1 B115	21
AB	DR15	DR15 Casa1 B615	33.3
AB	DR15	DR15 Fags1 B515	21.4
AB	DR15	DR15 Cora1 B315	30.8
AB	DR15	DR15 Algn1 B215	75
AB	DR15	DR15 Carb1 B415	64.3
AB	DR15	DR15 Quea1 B115	46.2
AC	DR15	DR15 Casa1 B615	12.9
AC	DR15	DR15 Fags1 B515	17.8
AC	DR15	DR15 Cora1 B315	96.9
AC	DR15	DR15 Algn1 B215	37
AC	DR15	DR15 Carb1 B415	99
AC	DR15	DR15 Quea1 B115	8.39
AD	DR15	DR15 Casa1 B615	95.1
AD	DR15	DR15 Fags1 B515	97
AD	DR15	DR15 Cora1 B315	98.0
AD	DR15	DR15 Algn1 B215	97.0
AD	DR15	DR15 Carb1 B415	99.3
AD	DR15	DR15 Quea1 B115	88.8
AE	DR15	DR15 Casa1 B615	11.8
AE	DR15	DR15 Fags1 B515	13.3
AE	DR15	DR15 Cora1 B315	87.5
AE	DR15	DR15 Algn1 B215	58.9
AE	DR15	DR15 Carb1 B415	100
AE	DR15	DR15 Quea1 B115	12.5
AF	DR15	DR15 Casa1 B615	35.7
AF	DR15	DR15 Fags1 B515	47.4
AF	DR15	DR15 Cora1 B315	72.8
AF	DR15	DR15 Algn1 B215	57.2
AF	DR15	DR15 Carb1 B415	94.8
AF	DR15	DR15 Quea1 B115	54.6
AG	DR15	DR15 Casa1 B615	61
AG	DR15	DR15 Fags1 B515	72.1
AG	DR15	DR15 Cora1 B315	93.9
AG	DR15	DR15 Algn1 B215	77.9
AG	DR15	DR15 Carb1 B415	86.8
AG	DR15	DR15 Quea1 B115	86.4
AH	DR15	DR15 Casa1 B615	75.9
AH	DR15	DR15 Fags1 B515	63.4
AH	DR15	DR15 Cora1 B315	89.6
AH	DR15	DR15 Algn1 B215	81.9
AH	DR15	DR15 Carb1 B415	97.3
AH	DR15	DR15 Quea1 B115	81.7

***Supplementary Table 3**: Bet v 1 and tree pollen homolog-specific CD4+ T cells (Cas a 1, Fag s 1, Que a 1, Aln g 1 and Cor a 1) were tracked in eight DR15:01-restricted birch pollen allergic individuals PBMCs. The percentage of co-stained T-cell populations was determined relative to Bet v 1-specific T cells and shown in table above.*
