# Supplementary material for: T cells specific to multiple Bet v 1 peptides are highly cross-reactive toward the corresponding peptides from the homologous group of tree pollens
Source: Front Immunol. 2023 Nov 22;14:1291666. doi: 10.3389/fimmu.2023.1291666 (PMC10702988; doi:10.3389/fimmu.2023.1291666)
Supplement: Supplementary file 12 [file DataSheet_1.docx]

# Supplementary Material

**Supplementary text:**

Supplementary text 1.1:

The data demonstrated in Figure S1 shows significant correlations between IgE reactivity towards birch and alder (top left), and hazel, (Top middle) as well as birch and oak (top right). The birch IgE cross-reactivity to Car b Fag g and Cas a was also evaluated and is shown at the bottom panel. The strongest significant correlation in IgE titres was seen between birch and alder and the weakest was between birch and beech. No correlation was observed between birch and chestnut. This is in line to our previous publication [17].

## Supplementary 1.2 Epitope mapping of group 1 allergens

We investigated the T-cell responses to individual peptides spanning the complete aa sequence of each of the four group 1 major allergens (Bet v 1, Aln g 1, Cor a 1, and Que a 1) to elucidate, if the homologous peptides contain important T-cell epitopes relevant for cross-reactivity as indicated by the responses to allergen extracts and purified group 1 allergens. The response frequency of the individual 20-mer peptides are aligned for Birch lines (Figure 3a ) and for Oak lines (Figure 3b) with the responses of individual donors and the strength of the responses depicted in the heat maps below each bar plot (Figure 3a and b). For birch T-cell lines almost all Bet v 1 peptides contained important T-cell epitopes with response frequencies close to or above 50% and only peptides #5_(41-60)_, #6_(51-70)_ and #13_(121-140)_, _#_14_(131-150)_ were recognized by a smaller subset of T-cell lines. A similar pattern of response frequencies was seen for Aln g 1 peptides but with lower response frequencies to peptide #1_(1-20)_ and #3_(21-40)_ and higher for peptide #13_(121-140), #_14_(131-150)_ than seen for the homologous peptides in Bet v 1. Stimulation with Cor a 1 peptides also resulted in T-cell activation for all peptides with peptides #2_(11-30)_, #11_(101-120),_ #12_(111-130)_, and #15_(141-159)_ around 50% and the rest of the peptides activating 20-40% of the T-cell lines. Response frequencies towards Que a 1 peptides were the lowest in general (0-30%). Peptides #5_(41-60)_ and #12-14_(111-150)_ did not activate any birch T-cell lines and peptides #3_(21-40)_ (24%), #10_(91-110)_ (28%) and especially #15_(141-159)_ (52%) contained important T-cell epitopes and the response frequency to Que a peptide #15_(141-159)_ was similar to the frequency of peptide #15_(141-159)_ in the other group 1 allergens investigated.

The response pattern and strength of the birch T-cell lines from individual donors shown in the lower part of each figure shows that most patients responded to multiple Bet v 1 peptides with one or two peptides reaching the maximal response for each donor and with a very diverse pattern from donor to donor. Similar patterns were seen for individual responses to Aln g 1 and Cor a 1 peptides with a slightly reduced intensity for most donors, and some donors showing only vague responses to a few peptides. For Que a 1 peptides the responses were limited to a few peptides in most of the birch T-cell lines generally responding vaguely and mainly peptide #15_(141-159)_ giving rise to medium and strong responses. The amino acid sequence of the Bet v 1 homologs and the location of different peptides evaluated in the current study are illustrated in supplementary Figure S6.

For oak T-cell lines (Figure 3b), several peptides reached a response frequency in the range of 50% and almost all lines responded to Que a 1 peptide #15_(141-159)_. Moreover, some lines even responded to Que a 1 peptides #5_(41-60)_#, #12_(111-130)_, and #14_(131-150)_, which were lacking recognition completely in the birch T-cell lines. A similar pattern of response frequencies was seen for Cor a 1, Aln g 1, and Bet v 1 peptides with some differences in the frequency of response to the individual peptides but in general high frequencies throughout the group of peptides investigated and with peptide #15_(141-159)_ reaching frequencies between 63% (Cor a 1) and 86% (Aln g 1).

The response pattern and strength of the oak T-cell lines shows that most individuals are responding to multiple peptides with more peptides reaching the maximal response than generally seen for the peptide responses in the birch T-cell lines. Patterns similar to responses to Que a 1 peptides were seen for individual responses to Cor a 1, Aln g 1 and also for Bet v 1 peptides with similar overall intensity for most donors but with responses to some peptides differing substantially from species to species for the individual donors. Again peptide #15_(141-159)_ gave rise to medium and strong responses in the majority of donors for all group 1 allergens investigated.
